# Towards point-of-care HIV testing: Terahertz PCF sensor integration and miniaturization

**DOI:** 10.1371/journal.pone.0327357

**Published:** 2025-07-01

**Authors:** Most. Momtahina Bani, Md. Mynuddin, Khalid Sifulla Noor, A. H. M. Iftekharul Ferdous, Md. Safiul Islam

**Affiliations:** Department of Electrical and Electronic Engineering, Pabna University of Science and Technology, Pabna, Bangladesh; CMRIT: CMR Institute of Technology, INDIA

## Abstract

The Human Immunodeficiency Virus, or HIV, is a retrovirus which aims at CD_4_ cells in particular to destroy the body’s natural defenses along with impair its capacity for fighting off illness plus ailments. In this circumstance we suggested a Photonic Crystal Fiber (PCF) sensor for detection of this lethal virus. Our intended sensor exhibits Max Relative Sensitivity (RS) 95.2% & 95% for x & y axis correspondingly when f = 3.0 THz. It also impresses us with minimal Confinement Loss (CL) of 1.06 × 10^−7^ & 2.04 × 10^-7^dB/m plus Effective Material Loss (EML) of 0.0078 & 0.0110 cm^−1^ in that order for x & y axis. Initial detection alongside prompt cures for HIV are made feasible by the remarkable efficacy of PCF biosensor observations in this regard. PCF gadgets offer rapid, non-invasive medical diagnosis, which simplifies the need for further evaluation & allows for an assessment of a condition’s progression as it progresses. Precise assessment may be beneficial when investigating advancements that improve worldwide identification and therapeutic choices. It is possible that the rapid identification of these harmful microbes was made possible by the remarkable identifying ability provided by the new PCF. In conclusion, healthcare offers a plethora of opportunity.

## 1. Introduction

The ultimate in optics development is the photonic crystal fibre (PCF), whose complex structure of a carefully organized matrix of air pockets has revolutionized transmitting light. Those micro-structured cores enable unmatched adaptability along with utility via regulated control of illumination, thereby launching a new chapter in optical transmission & imaging exploration. Because of its distinct framework, PCF can have customized scatter characteristics, which has led to an array of bandwidth light bulbs for spectral analysis, scattering-compensating fibres, and superfast pulses [1,2]. Furthermore, its capacity to contain information in minuscule air gaps promotes improved regressive impacts enabling uses in frequencies switching, supercontinuum the next, and nonlinear optical technology. PCF sees several uses in fields that include biology to connectivity, propelling developments like high-power beams of light, fiber-optic sensors, even medical visualization. The coming years promises further creativity & influence within a variety of disciplines as it explores exclusive capabilities plus uses & extends beyond the limits of PCF hardware [3,4]. The band of frequencies spanning microwaves alongside infrared rays contains THz energy which has attracted considerable interest from a variety of academic fields because of its special qualities and broad range of uses. The energy it emits has unidirectional properties and can be safely used for photography & monitoring purposes in science and medicine. Their spectra range from 0.1 to 10 THz. Its versatility in penetrating fabrics, newspaper, & polymers makes it easier for inspection in addition safety checks in sectors like aircraft & pharma. Additionally, THz energies are sensitive to rotate alongside structural oscillations in molecules, making spectral analysis useful for pharmacological assurance along with biochemical recognition. THz waves has the possibility to revolutionize new wireless networks providing unparalleled storage by enabling ultra-high-speed information exchange. THz pulses were difficult to generate, identify, as well as manipulate, but development is still being done to fully realize their promise and create novel innovations that will revolutionize everything including telecoms to medicine [5–9].

Through the use of the fiber’s heat awareness, PCF devices are capable to identify variations in temperatures and convert these into quantifiable shifts in the speed of photon passage. PCF gauges are used in a variety of industries, including ecological tracking & factory surveillance, because of their superior sensitivity as well as rapid responses [10,11]. Utilizing the special qualities of the fibre, PCF devices in gauging pressure possess the ability to identify alterations to spectral properties brought on by stress and convert these into outputs that can be measured. These devices, which have superior sensing & precision, are used in the automobile as well as aviation sectors [12,13]. PCF indicators convert fluctuations in the amount of water into discernible shifts in wavelength by taking use of the fiber’s susceptibility to those variations. These instruments are used in HVAC devices for accurate moisture measurement, chemical oversight, & monitoring the climate due to their great precision as well as dependability [14,15]. The customized characteristics of PCF fibres are used by chemical identification PCF monitors to combine with certain compounds and produce measurable shifts in how light travels. Having outstanding sensitivity & selectivity, the presence plus level of particular chemicals might be ascertained by examining those modifications [16,17]. Utilizing the special features of the fibre, PCF devices enabling alcoholic testing connect to alcoholic beverages to cause quantifiable variations in spectral characteristics. Alcohol identification may be done quickly and precisely via studying the ocular reflexes of PCF instruments, which helps to promote safe behavior across a variety of contexts. Development is still being done to improve sensor efficiency while producing affordable, mobile technologies for the broad demand on alcohol recognition [18,19]. PCF detectors over blood element identification use the special characteristics of the fibre in conjunction with certain blood components and produce photons that are easily distinguished. Continuous surveillance and evaluation of blood factors may be accomplished by examining the visible reactions of PCF instruments. This allows for swift action with individualized treatment [20,21]. With their capacity to identify tumour biomarkers, examine migrating malignant cells, assess tissue anatomy, and facilitate surgical evaluation, PCF monitors have excellent prospects for cancer diagnosis. Sustained progress in PCF detection devices, in conjunction with continuous investigation into cancer genetics therefore tests, could potentially augment early identification, ameliorate clinical outcomes, and finally diminish the global cancer load [22,23]. By utilizing the special qualities of the fibre that bond between specific gas particles and cause observable shifts in spectral properties, PCF instruments are used in gas identification. It is possible to identify gases accurately and quickly by examining the visual replies of PCF detectors. This allows for quick identification of dangerous gases leaking or harmful emissions [24,25]. The potential of PCF monitors for drug identification is evident in their capacity to identify specific substances, track metabolic alterations, and evaluate the medicinal value of drugs. Improvements in drug testing, surveillance, overall investigation could be made possible by further developments in PCF detector technology & current research into drug detection techniques. These developments could support safety and health protection programs [26,27]. Using customized bonds to targets, PCF devices identify petrochemicals quickly as well as accurately, making them useful for ecological tracking and safety in factory purposes [28,29].

The suggested PCF instruments may be made operational by attaching particular molecules as well antibody to HIV antigens or antibodies found in body fluids. HIV antigens or antibodies may trigger detectable alterations within a customized PCF detector’s transparency, that are then examined to determine if HIV is present or not. While it hasn’t been used much nonetheless, the technique shows hope in precise and on-time HIV testing, supporting preventive care and tracking initiatives. The working principle of the proposed PCF sensor relies on the interaction between guided terahertz light and HIV-specific analytes present in the core. When the analyte binds to functionalized regions, it induces a local refractive index change, modifying the effective index and optical confinement. This alteration affects key parameters such as confinement loss and relative sensitivity. Light–analyte interactions are thus translated into measurable spectral shifts, enabling precise HIV detection.

Now, in this research we also investigate other important properties of this suggested sensor. For example the NA for this detector are 0.313 & 0.283 for x and y axis respectively. In addition the EA for this PCF are 2.91 × 10^−8^ m^2^ & 3.64 × 10^−8^ m^2^ for x and y axis. Early identification of HIV indicators in human tissue is made possible by this PCF sensors’ excellent receptivity along with rapid responses. Their propensity for miniaturization makes laboratory testing easier, and its precise identification skills reduce accidental positives and rejections. Relevance in HIV diagnoses is expanded by acceptance having complicated specimens which can be incorporated within mobile devices, which enables decentralized testing—critical in environments having scarce assets.

## 2. Methodology

Designing and specifying the PCF architecture’s dimensions and constituents is the initial phase in using COMSOL Multiphysics to create a PCF biosensor. Using COMSOL Multiphysics for HIV detection with PCFs, the simulation models the mass transfer phenomenon with biochemical responses involving HIV proteins as well as the outermost functioning of the PCF. We construct this unique detector and study it using COMSOL MULTIPHYSICS software. This PCF has a unique hybrid coating and an innovative rectangular core in addition 20 varying shape air holes are created in the cladding. The design choice of incorporating 20 air holes in the cladding region was the outcome of a meticulous optimization process conducted through iterative simulations. Each hole’s size, shape, and position were strategically adjusted to achieve optimal light confinement while preserving the structural symmetry of the photonic crystal fiber. This parameter selection process involved carefully balancing the trade-offs between maximizing the Relative Sensitivity and minimizing the Confinement and Effective Material Loss. By using a systematic approach rather than trial-and-error, we ensured that the sensor design is both theoretically efficient and practically viable, offering high performance in real-world applications. The suggested model’s foundation substance is Zeonex. The computed RI for Zeonex is RI = 1.53. Zeonex is the preferred choice for PCF sensor for a variety of reasons. Initially, the fiber core’s strong light confinement is a result of low refractive index that is crucial for detection of minor RI changes that are caused by the presence of HIV. Furthermore, Zeonex’s exceptional thermal stability guarantees consistent sensor performance by mitigating the consequences of temperature fluctuations. Zeonex is widely preferred in biomedical and THz waveguide designs due to its low dielectric loss, high optical transparency in the THz region, and exceptional moldability, making it ideal for precise fabrication. Additionally, its compatibility with biological samples and its stability under harsh environmental conditions enhance its suitability for point-of-care diagnostic applications. Zeonex’s properties make it particularly suitable for terahertz photonic applications. These include its low water absorption, high dimensional stability, and low birefringence, all of which contribute to maintaining consistent performance in varying environmental conditions, such as fluctuating temperature and humidity. Additionally, Zeonex exhibits excellent optical transmission in the 0.1–3 THz range, crucial for achieving optimal sensor sensitivity. Its biocompatibility is another key factor, allowing direct contact with biological fluids, which is especially important for applications like HIV sensing, where interaction with biological samples is essential for accurate detection. The optical properties of Zeonex play a pivotal role in enhancing the performance of our sensor. With a low refractive index (~1.53), Zeonex creates a significant index contrast with air, which improves the mode confinement within the core of the photonic crystal fiber. This enhanced confinement facilitates stronger light-matter interactions, leading to an increased sensitivity to refractive index (RI) changes caused by the presence of HIV biomarkers. In our revised manuscript, we have included a dedicated section that highlights how Zeonex’s optical advantages—such as its low refractive index and excellent transparency—directly contribute to the sensor’s high sensitivity, enabling accurate detection of biological markers. These attributes reinforce the sensor’s effectiveness in real-world HIV detection applications.

**Fig 1:**
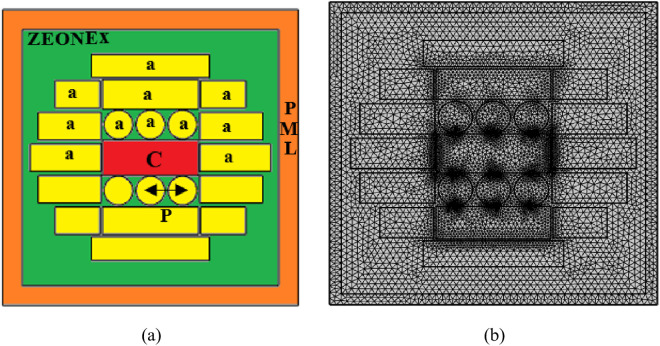
(a) Exhibit our invented detector’s cross-section, (b) Exhibit mesh sight of our invented PCF’s cross-section.

It is optimal for THz-based sensing due to its minimal absorption loss in the terahertz (THz) range, which enables improved signal transmission and increased sensitivity. Zeonex is also suitable for reliable, high-sensitivity biomedical applications such as HIV detection due to its biocompatibility and mechanical stability. Although our study primarily relies on numerical simulations, we have now discussed the feasibility of fabricating Zeonex-based photonic crystal fibers (PCFs) using established techniques such as the stack-and-draw method and 3D extrusion. These fabrication methods are well-documented and widely used for producing PCFs with precise control over geometry and material properties. By exploring these practical approaches, we aim to demonstrate that our proposed sensor geometry is not purely theoretical but can be realistically fabricated and tested. This addition strengthens the practical relevance of our work and paves the way for future experimental validation.This time, Fig 1a depicts cross-section for this detector. PML excels at photonic simulations, especially for PCF sensors. Its key benefit is that it works as an artificial absorption border to reduce simulation domain edge reflections. The PML simulates an endless environment by absorbing outgoing waves, preventing undesired reflections from interfering with the core simulation. In applications that need containment and transmission, electromagnetic wave propagation can be accurately modelled. By decreasing boundary-induced artifacts, a PML improves PCF detector CL, sensitivity, and EML. PMLs increase simulation precision and dependability, which is crucial for enhancing sensor performance in real-world applications.

The term “mesh” in the context of PCF sensing implies the separation of the geometrical makeup of the PCF into fragments or cells, allowing computations in programs such as COMSOL Multiphysics. By splitting the PCF’s area, this mesh makes it easier to calculate physical properties including photon transmission, reactions with chemicals, and detecting warnings, guaranteeing precise simulation as well as evaluation of the instrument. Mesh provides. A total of 92 convex stages; 1515 boundary units in addition to 12676 elements; minimum attribute rating: 0.4286. where the mesh arrangement according to the new PCF is displayed in Fig 1b.

**Fig 2:**
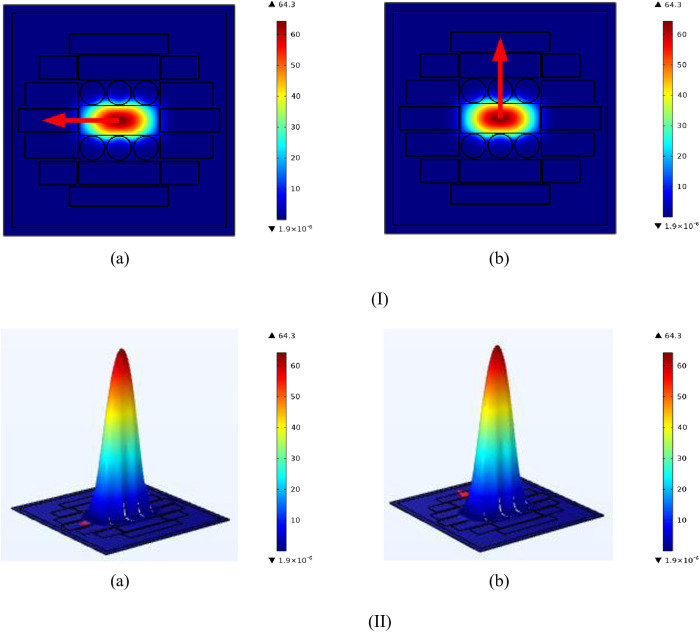
(I) Exhibit power distribution for (a) x-axis (b) y-axis and (II): Exhibit density distribution for (a) x-axis (b) y-axis.

Power distribution in PCF describes how optical energy is distributed and managed within the fibre’s structural core. The optical bandgap characteristics of the fiber regulate this distribution, enabling accurate control over light confinement and dispersion. Because of its distinct design, PCF can transmit among manipulate optical signals with great efficiency, which makes it useful for asymmetric optics, detecting, and telecom purposes. PCF’s power distribution is essential for maximizing signal integrity and enabling a range of functions in optical systems. Fig 2I Portray how power is distributed.

The term “density distribution” in our study specifically refers to the spatial distribution of the refractive index within the photonic crystal fiber (PCF) core and cladding structure. This refractive index profile is shaped by the geometric arrangement and dimensions of the air holes as well as the core material properties. Such distribution plays a crucial role in determining the optical mode shape, dispersion characteristics, and confinement behavior of the guided THz waves. Effective refractive index profile is resolved by this distribution, which affects modal dispersion and confinement, two important aspects of light propagation. PCF designers may engineer certain optical characteristics, like customized dispersion, nonlinear effects, and modal properties, which are essential for high-power pulse shipping, detecting, and telecommunication programs, by manipulating the density distribution. Enhanced functions in optical communication and photonics are made possible by the exact management of light made possible by the complex material composition inside the PCF core. Fig 2II density is distributed.

To simulate THz wave propagation, FEM and PML boundary conditions were used, but experienced challenges. The problem was accurately modelling THz waves’ interaction with the hollow-core PCF’s complex structure, especially the rectangular core and air aperture shape. The PML borders must absorb outgoing waves without reflections to prevent interference with wave propagation within the fiber. Additionally, FEM simulations required fine-tuning mesh resolution for accurate findings without computing overhead, as THz waves are particularly sensitive to fiber geometry. Accurate PML layer calibration was crucial to avoid numerical artifacts at the boundary from affecting simulation results. Although faced with challenges, the simulation settings were optimized to accurately forecast wave behaviour and sensor performance in the THz frequencies. While our sensor demonstrates excellent numerical performance, experimental validation remains absent due to the lack of cleanroom facilities and THz measurement equipment. Nonetheless, our FEM-based COMSOL simulations with PML boundaries offer reliable performance insights. This limitation is acknowledged in the conclusion, and future work will focus on collaborating with experimental labs to fabricate the Zeonex-based structure and validate the results under practical conditions.

## 3. Results and analyses

PCF geometry is separated into small components using the FEM for PCF. Using a computational approach, this procedure analyzes the equations developed by Maxwell to evaluate the transmission of light inside the PCF. To correctly convey the qualities related to light confinement including transmission, it takes into account geometrical aspects and the RI distribution. The scattering features, mechanical buildings, and electromagnetic circulation of PCFs are calculated using FEM. It makes it possible to analyze PCF layouts in great depth, taking into account the impact of flaws and attributes of the material. For a variety of uses, including monitoring, asymmetric optical science, among internet access, FEM helps optimize PCF architectures. This technique sheds light on PCF dispersal impacts leaking, especially phase interaction. PCFs with customized spectral characteristics are easier to envision along with manufacture to FEM computations. The detection mechanism of our proposed PCF sensor is based on the principle of refractive index (RI) sensing, where the presence of specific biomarkers like HIV antigens or antibodies alters the RI in the fiber core. This change influences the light propagation and is reflected in variations in parameters such as Relative Sensitivity (RS), Effective Material Loss (EML), and Confinement Loss (CL). To enable selectivity, the sensor’s internal surface can be functionalized with HIV-specific antibodies. Upon interaction with antigens present in bodily fluids, such as saliva or blood plasma, localized RI shifts occur due to molecular binding. These variations are detected with high accuracy because of the strong field confinement in the PCF core. Such RI modulation allows the sensor to specifically differentiate HIV-related analytes from other biomolecules. Our parameter optimization process was carried out through a series of simulations in COMSOL Multiphysics, where we systematically varied key parameters such as the pitch, air hole dimensions, and core geometry. Each variation was analyzed to assess its impact on critical performance metrics, including Relative Sensitivity (RS), Confinement Loss (CL), and Effective Material Loss (EML). The final geometry was chosen after identifying the optimal performance region, ensuring that the design was based on a systematic and reproducible approach, rather than arbitrary selection. This rigorous methodology underscores the reliability and robustness of our sensor design.

The term RS in PCF describes how a certain characteristic, including bandwidth or RI, changes in response to exterior stimulus information, such as pressure or heat. It quantifies the degree to which the stimulus-induced shift affects the variable of relevance. Whenever applications for detecting PCF are used, RS is essential because it allows minor variations in external factors to be recognized through tracking changes in the optical characteristics of the fiber. Enhanced adaptability, denoted by an elevated RS, increases the sensitivity of PCF-based sensing across a variety of measures, including temps, pressures, and biological substances. Gaining knowledge of RS enables PCF layouts to be optimized for particular sensory needs, enhancing their usability as well as generalizability.

Utilize these formulas based on math to figure out RS [30].


$$r=\frac{n_{r}}{n_{eff}}\times p\mathrm{\backslash nonumber}\;\%$$ (1)

Notwithstanding, P has an authentic function in recognizing an RI constituent.


$$\;p=\frac{\int_{sample}R_{e}\left(E_{x}H_{y}-E_{y}H_{x}\right)dxdy}{\int_{total}R_{e}\left(E_{x}H_{y}-E_{y}H_{x}\right)dxdy}$$ (2)

In comparison, E_x_ denotes attributes in an EF plane while H_x_ denotes substances in an MF plane.

**Fig 3:**
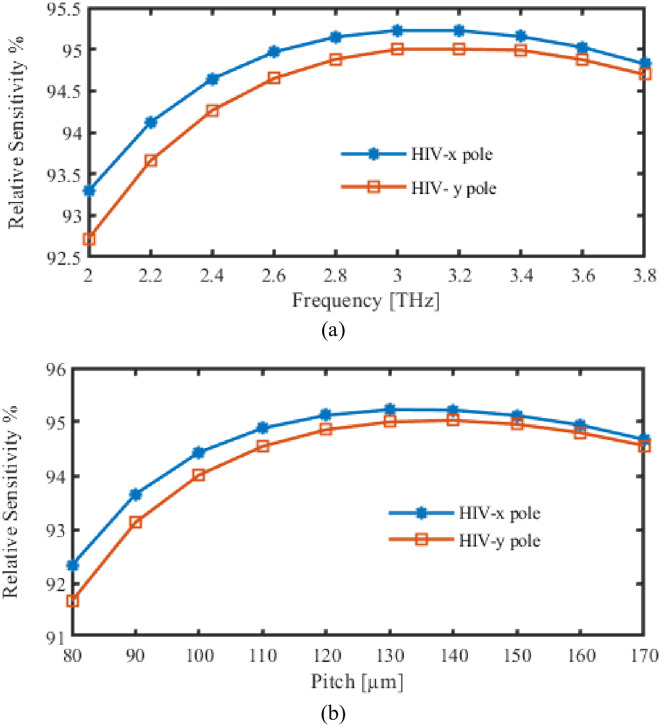
Display the effects of RS depending on the variations of (a) Frequency [THz] (b) Pitch [µm].

At first, we looked over RS concurrently as an indicator of pitch and frequency. Changes inside RS are shown in Fig 3a as an oscillatory variation spanning 2 and 3.8 terahertz. Conversely, Fig 3b demonstrates how the Pitch may affect the variety inside RS.

The graph of RS exhibits a trend of increasing frequency until approximately 3 THz, at which point it reaches a plateau. This trend is due to the fact that the interaction between the analyte (in this instance, the HIV virus or the medium in which it is present) and the guided light is weaker at lower frequencies, which leads to a decrease in sensitivity. Interplay between light field and analyte is further enhanced by the shortening of the wavelength as the frequency increases. The plateau suggests that the sensitivity has reached a maximum point as a result of the confined mode’s optimization of its overlap with the analyte.

Inside, material absorption, and dispersal, along with other inefficiencies within the PCF lattice can cause light to propagate more slowly. This phenomenon is known as EML. It calculates how well light travels along the fibre collectively. Architectural flaws, the nature of the material, plus manufacturing methods are some of parameters that affect EML. For PCFs to operate well across diverse programs, including asymmetric optical science, detection, along telecommuting, EML must be kept to a minimum. PCF functionality is improved and EML is reduced by using improved layouts in addition to resource selections.

Utilize these formulas based on math to figure out EML [30].


$$\alpha_{eff}=\frac{({\frac{\varepsilon_{0}}{\mu_{0}})}^{\frac{1}{2}}\;\int_{A_{\max}}n\alpha_{mat}{\left|E\right|}^{2}dA}{2\int_{ALL}S_{z}dA}$$ (3)

Where the loss coefficient of zeonex is field adjacent to $\alpha_{mat}$ and E is E.field.

**Fig 4:**
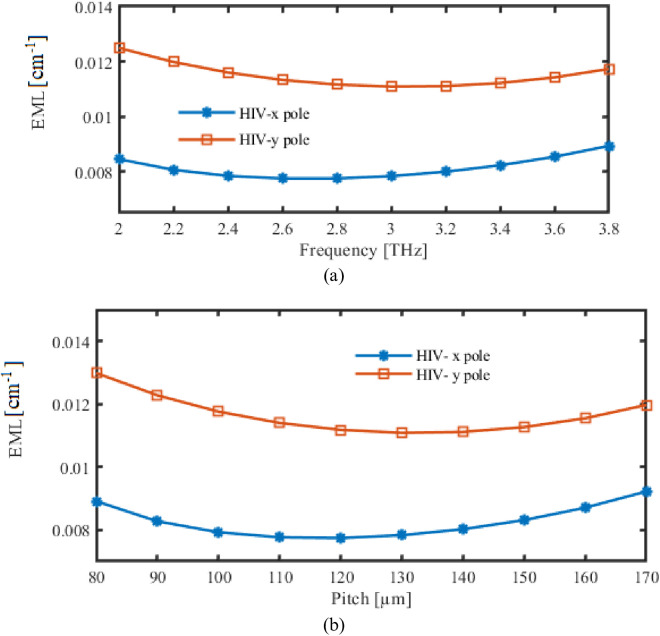
Display the effects of EML depending on the variations of (a) Frequency [THz] (b) Pitch [µm].

For our proposed PCF, we’ll immediately start looking through the EML. The relationship between EML, as well as the gadget’s frequency and pitch, is displayed in Fig 4. In this section, difference in EML during functioning FR variations is seen in Fig 4a. Conversely, EML fluctuation is shown in Fig 4b as the pitch changes.

The material’s properties are reflected in the minor decreasing trend of the EML graph with frequency. The material utilized, Zeonex, exhibits minimal loss in the THz region. As the frequency increases, the material’s inherent absorption loss decreases marginally, leading to a decrease in EML.

By propagating inside the fibre core but escaping because of flaws or abnormalities in the photonic crystal lattice, light particles in PCF experience CL, which results in dispersion alongside the loss of power. It has a significant impact on PCF functionality plus transport efficiency, especially at elevated wavelengths as well as across vast distances. To minimize CL, the PCF’s structure and layout must be optimized to improve photon containment inside the core, minimize spillage, along maximize propagation capacities. Detailed manipulation of the optical crystallization is usually required for this improvement to reduce flaws while boosting lighting steering inside the fibre core.

Utilize these formulas based on math to figure out CL [30].


$$L_{c}=\frac{40\pi }{\ln\left(10\right)\lambda }img\left(n_{eff}\right)\times \frac{10^{6}dB}{m}$$ (4)

Additionally, the visualization of n_eff_ represents the EMI region that is shown, and λ represents a true spectrum.

**Fig 5:**
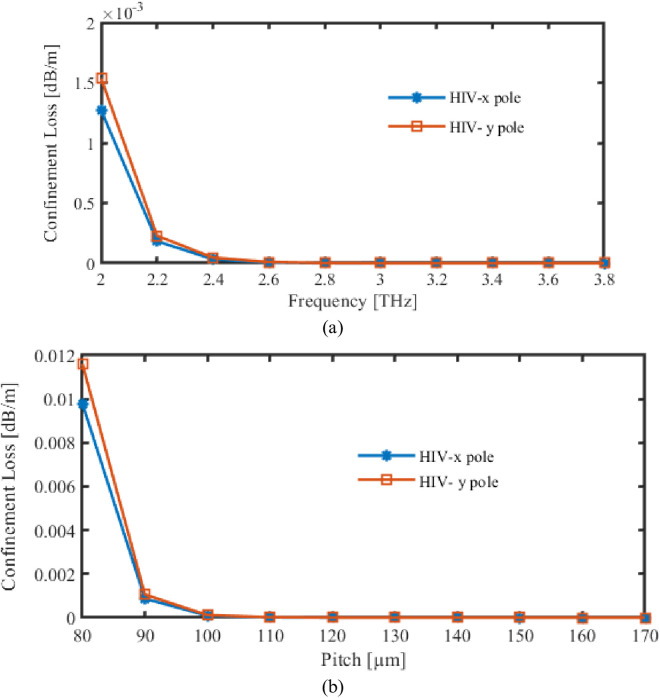
Display the effects of CL depending on the variations of (a) Frequency [THz] (b) Pitch [µm].

Within Fig 5, CL of this suggested recognition while different frequencies as well as pitch fluctuations is showcased. In the case in point, Fig 5a demonstrates the CL during carrying out FR fluctuations. Variation in CL regarding pitch characteristic modifications is displayed in Fig 5b.

Initially, CL experiences a significant reduction as the frequency increases, but it subsequently stabilizes at higher frequencies. This is due to the fact that the light is less densely constrained within the core at lower frequencies, resulting in a greater amount of energy escaping into the cladding and resulting in higher losses. The loss is reduced as the mode becomes more confined to core as FRs augmentations.

In PCF, NA is a measurement of the fibre’s radiation-gathering capacity, which establishes its capacity to absorb light from the surrounding medium. It is affected by PCF’s construction and difference in RI that exists between cladding and core components. Greater light-capturing capabilities and higher light-gathering performance are indicated by greater NA values. In PCFs, NA is frequently manipulated precisely to produce specified spectral characteristics because of the architecture of the air-hole structure inside the fibre. Technologies like sensors in electronics followed by information technology that need appropriate optical coupling must comprehend along with optimized NA.

Utilize these formulas based on math to figure out NA [31].


$$\;NA=\frac{1}{\sqrt{1+\frac{{\pi A}_{eff}f^{2}}{c^{2}}}}\approx \frac{1}{\sqrt{1+\frac{{\pi A}_{eff}}{\lambda^{2}}}}$$ (5)

Utility wavelengths are represented by λ, while the PCF’s EA is displayed by A_eff_.

**Fig 6:**
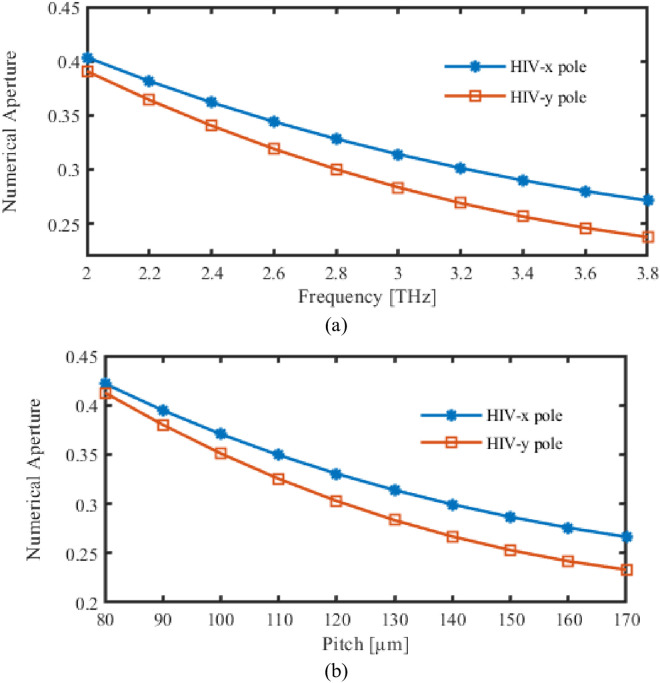
Display the effects of NA depending on the variations of (a) Frequency [THz] (b) Pitch [µm].

By using Fig (6), NA for the recommended PCF is analyzed while pitch as well as FRs are tweaked. Variations in frequencies result in fluctuations in EA as displayed in Fig 6a. But Fig 6b highlighted how EA altered as suggested PCF was altered.

The NA is sufficiently high at 3 THz and 130 µm pitch to effectively gather light while still maintaining confinement within the core. This balanced NA is an excellent choice for ensuring that light interacts effectively with the analyte without causing excessive divergence, thereby increasing sensitivity to the presence of HIV.

The photonic energy distribution throughout the fibre core is represented by the EA in PCF. It is a significant component that affects scattering capabilities including a variety of complex phenomena. Since the photonic crystal arrangement in PCF allows for special steering techniques, EA is frequently bigger than in standard fibres. Enhanced photon concentration within the material’s core, which lowers ripples and thus improves the clarity of the signal, is implied by a bigger EA. The RI characteristics, loop geometry, central dimension, along with other PCF specifications are adjusted by scientists and technicians to modify EA including customizing the spectral properties of the fibre for nonlinear imaging as well as high-power laser shipping, among other uses. To maximize PCF-based photonic technologies’ effectiveness and reliability, EA must be understood alongside optimization.

Utilize these formulas based on math to figure out EA [32].


$$A_{eff}=\;\frac{{[\int I\left(r\right)rdr]}^{2}}{{[\int I^{2}\left(r\right)rdr]}^{2}}$$ (6)

Consequently, the E.field is displayed over the identifying component when I(r) = |E|^2^.

**Fig 7:**
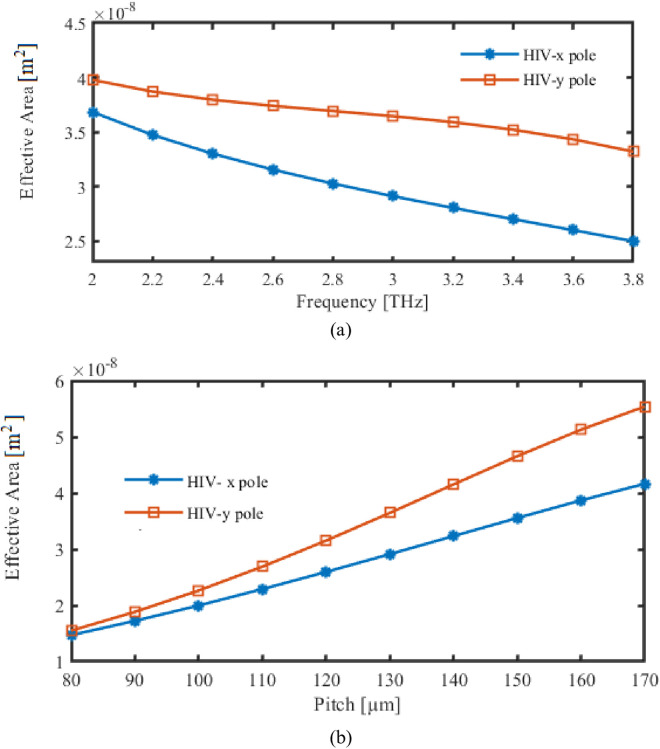
Display the effects of EA depending on the variations of (a) Frequency [THz] (b) Pitch [µm].

The analysis that follows will examine how the EA changed in response to changes in the frequencies that operated the pitch of the PCF. Here, the correlation between EA as well as frequency appears in Fig 7a. EA varies in tandem with alterations in FR. Pitch and EA are related, as Fig 7b illustrates. Pitch adjustments cause similar fluctuations in EA.

EA is relatively small at 3 THz and 130 µm, which guarantees a strong confinement of light within core and enhances the interaction with HIV analyte. Electromagnetic field is concentrated in a reduced effective area that improves sensitivity to variations in RI of analyte.

As a measure of the geographic area of the visible light area, the circumference of the visible pattern contained inside the fibre core is designated to as spot size in sensor. It is affected by variables, including wavelengths, RI contrary, and fibre architecture. Because of the special optical crystal arrangement in PCFs, the spot size may be precisely controlled, making customization possible for particular uses. More visual brightness plus greater lighting containment are indicated by a decrease in spot size, which is advantageous with highly sensitive applications. To satisfy quality requirements in industries such as sensors, laser infrastructure, as well as telecommuting, engineers optimize spot size by modifying elements including central dimension, air-hole shape, and RI profile. In a variety of photonic programs, optimizing PCF effectiveness and efficacy requires a consciousness of and oversight of spot size.

Utilize these formulas based on math to figure out spot size [33].


$$W_{eff}=R\times \left(\mathrm{.65}\times {1.619\times V}^{-1.5}+{2.789\times V}^{-6}\right)$$ (7)

When using the altered FR band V that has a rectangular core width R.

**Fig 8:**
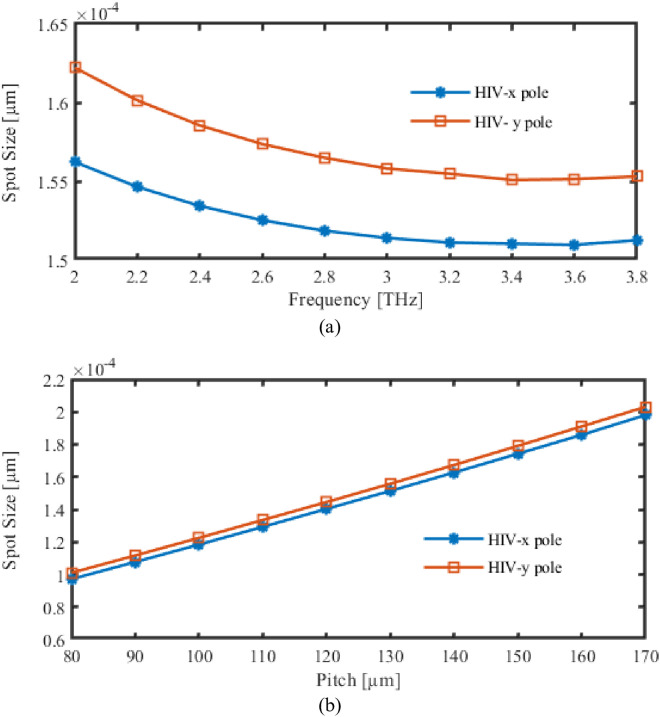
Display the effects of spot size depending on the variations of (a) Frequency [THz] (b) Pitch [µm].

Fig 8 illustrates the correlation between pitch, frequency response (FR), and spot size. The size of the spot is illustrated in Fig 8a, indicating that it varies with the corresponding FR. Conversely, the influence of the recommended PCF’s pitch on its placement is depicted in Fig 8b.

Spot size decreases with frequency until approximately 3 THz, at which point it begins to marginally increase. Initially, the wavelength decreases as FRs increases, and mode is more tightly confined to core, resulting in a smaller patch size. However, the increased frequency may result in a modest increase in spot size as a result of mode-field adjustments beyond a certain threshold.

When light propagates through a PCF, the total loss includes all of the elements that contribute to transmission degradation. This covers both extrinsic losses involving splicing along with connection losses as well as internal losses such as substance absorption alongside dispersion. Furthermore, because of their special framework, PCFs are susceptible to particular losses including confining loss and bent loss. In PCF-based structures, reducing total loss is essential to preserving the confidentiality of signals and also optimizing the speed of transmission. For purposes that include high-power pulse supply to telecommuting, designers use a variety of strategies, such as choosing materials, and optimized fibre layout, thus cautious dealing with, to reduce loss while enhancing system efficiency as a whole.

Utilize these formulas based on math to figure out total loss [30].


$$Total\;Loss\;=\;\alpha_{eff}+L_{c}$$ (8)

Therefore, $\alpha_{eff}$ is EML and $L_{c}$ is CL.

**Fig 9:**
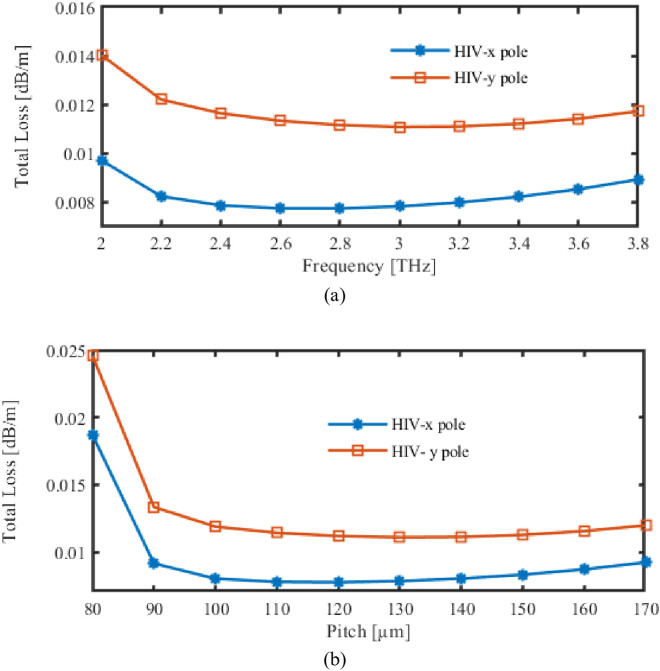
Display the effects of total loss depending on the variations of (a) Frequency [THz] (b) Pitch [µm].

In this instance, Fig (9) illustrates not only the overall loss but also the correlation between pitch and FR. Fig 9a will display the overall loss and illustrate how the loss varies with adjustments in the operating FR. However, Fig 9b depicts how pitch of suggested PCF affected where it was placed.

The total loss graph is a composite of material and confinement losses. It commences at a high level at lower frequencies, diminishes as the frequency increases, and subsequently stabilizes, a phenomenon that is comparable to confinement loss trends. The reduction in light leakage from the core is a result of enhanced mode confinement at higher frequencies.

In PCF, the term “birefringence” characterizes a scenario wherein photons in the fibre encounter two distinct refractive factors across opposing polarized orientations. This is caused by the intrinsic architectural anisotropic or imbalance of PCF concepts, which includes the existence of air-filled pockets placed in particular configurations. PCF’s birefringence can be designed and adjusted to meet the needs of a variety of uses, including orientation-maintaining fibres for instruments or equipment that are dependent on polarity. Developing equipment that depends on polarization management, including optical detectors, communication networks, and interfacing devices, requires a knowledge of as well as the ability to manipulate birefringence in PCF.

Utilize these formulas based on math to figure out birefringence [30].


$$B=\;\left|n_{x}-\;n_{y}\right|$$ (9)

This suggests that birefringence is defined as entire difference between the effective RIs of x and y-oriented portions.

**Fig 10:**
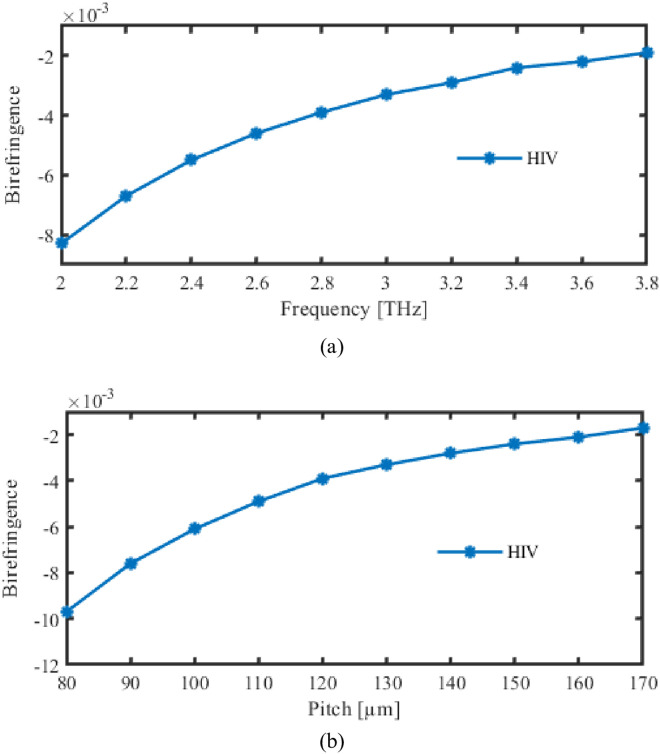
Display the effects of birefringence depending on the variations of (a) Frequency [THz] (b) Pitch [µm].

Birefringence of the suggested photonic crystal fiber is analysed by varying the pitch and frequency, as seen in Fig 10. The variation towards birefringence as the frequency varies is shown in Fig 6 (10). However, Fig 6 (10) demonstrated changes in birefringence during pitch as the suggested PCF was changed.

Stable birefringence is desired in this application at 3 THz and 130 µm because it enables consistent mode separation and reliable measurement across the operational frequency, thereby enabling the precise capture of the refractive index changes caused by HIV.

We had an organized conversation about this desired detector in addition to PCF screens that are in use now. Together with our PCF outcomes, Table 1 provides a summary of main elements of previous PCF. There was an older version made with the express purpose of building PCF measuring tools. Usually, silicon dioxide and a comparable substance are combined to create the initial form. A prevalent technique for embellishing stacking objects involves reheating the container and thereafter dragging it down a little quantity of fiber while preserving its original shape. Scanning partners can work together to provide particular features by carving or engraving apertures. Enabling useful compounds that look great entails increasing selection and then sensitivities. Regarding particular detectors, it is imperative to incorporate detecting techniques, such as microscopic particles that inhabit the interiors of cells [37,38].

**Table 1:** Exhibit a relative differentiation among the feature between the existing as well suggested sensors.

Rf.	Analyte	Fr (THz)	Rs (%)		CL (dB/m)	EML (cm^-1^)	NA
[31]	Ethanol	1.0	68.87		7.79 × 10^-12^	0.05	0.356
[32]	Formalin	1.8	77.71		2.79 × 10^-11^ (cm^-1^)	0.0048	–
[33]	Hemoglobin	1.5	80.56		1.13 × 10^-14^ (cm^-1^)	–	–
[34]	Cyanide	2.0	85.8		1.62×10^−9^	0.00859	–
[35]	Blood Cell	2.0	87.68		3.09×10^–17^	–	–
[36]	Alcohol	1.2	93.3		9.80 × 10^-18^	–	–
Our Sensor	HIV	3.0	X axis	95.20	1.06 × 10^−7^	0.0078	0.313
			Y axis	95.00	2.04 × 10^−7^	0.0110	0.283

## 4. Conclusion

The Human Immunodeficiency Virus, or HIV, is a retrovirus which aims at CD4 cells in particular to destroy the body’s natural defenses, along with impairing its capacity for fighting off illness plus ailments. In this circumstance we suggested a PCF sensor for the detection of this lethal virus. Initial detection alongside prompt cures for HIV are made feasible by the remarkable efficacy of PCF biosensor observations in this regard. PCF gadgets offer rapid, non-invasive medical diagnosis, which simplifies the need for further evaluation & allows for an assessment of a condition’s progression as it progresses. we suggested a Photonic Crystal Fiber (PCF) sensor for the detection of this lethal virus. Our intended sensor exhibits Max Relative Sensitivity (RS) 95.2% & 95% for x & y axis correspondingly when f = 3.0 THz. Designing and specifying the PCF architecture’s dimensions and constituents is the initial phase in using COMSOL Multiphysics to create a PCF biosensor. Using COMSOL Multiphysics for HIV detection with PCFs, the simulation models the mass transfer phenomenon with biochemical responses involving HIV proteins as well as the outermost functioning of the PCF. The proposed design is compatible with emerging mobile and wearable diagnostic tools due to its compact geometry and low loss. Furthermore, we highlighted the practical considerations such as surface functionalization with antibodies, real-sample testing, and challenges in field deployment like calibration, environmental interference, and signal processing. These additions demonstrate a forward-looking perspective aimed at practical and scalable deployment.

## Supporting information

S1 File Data sheet. (PDF)
