# Supplementary material for: Towards point-of-care HIV testing: Terahertz PCF sensor integration and miniaturization
Source: PLoS One. 2025 Jul 1;20(7):e0327357. doi: 10.1371/journal.pone.0327357 (PMC12212498; doi:10.1371/journal.pone.0327357)
Supplement: S1 File — (PDF) [file pone.0327357.s001.pdf]

wave	freq	p	nr	l	aff	real-x pol
1.50E-04	2.00E+12	1.30E-04	1.42	200	0.985	1.34410000000000
1.36E-04	2.20E+12	1.30E-04	1.42	210	0.985	1.35550000000000
1.25E-04	2.40E+12	1.30E-04	1.42	220	0.985	1.36460000000000
1.15E-04	2.60E+12	1.30E-04	1.42	230	0.985	1.37200000000000
1.07E-04	2.80E+12	1.30E-04	1.42	240	0.985	1.37810000000000
1.00E-04	3.00E+12	1.30E-04	1.42	250	0.985	1.38320000000000
9.38E-05	3.20E+12	1.30E-04	1.42	260	0.985	1.38750000000000
8.82E-05	3.40E+12	1.30E-04	1.42	270	0.985	1.39120000000000
8.33E-05	3.60E+12	1.30E-04	1.42	280	0.985	1.39430000000000
7.89E-05	3.80E+12	1.30E-04	1.42	290	0.985	1.39710000000000
1.00E-04	3.00E+12	8.00E-05	1.42	200	0.985	1.33340000000000
1.00E-04	3.00E+12	9.00E-05	1.42	210	0.985	1.34880000000000
1.00E-04	3.00E+12	1.00E-04	1.42	220	0.985	1.36060000000000
1.00E-04	3.00E+12	1.10E-04	1.42	230	0.985	1.36990000000000
1.00E-04	3.00E+12	1.20E-04	1.42	240	0.985	1.37730000000000
1.00E-04	3.00E+12	1.30E-04	1.42	250	0.985	1.38320000000000
1.00E-04	3.00E+12	1.40E-04	1.42	260	0.985	1.38810000000000
1.00E-04	3.00E+12	1.50E-04	1.42	270	0.985	1.39220000000000
1.00E-04	3.00E+12	1.60E-04	1.42	280	0.985	1.39560000000000
1.00E-04	3.00E+12	1.70E-04	1.42	290	0.985	1.39860000000000

real-y pol	imag-x pol	imag-y pol
1.35240000000000	3.4881000000000E-09	4.2217000000000E-09
1.36220000000000	4.5544000000000E-10	5.6976000000000E-10
1.37010000000000	7.4612000000000E-11	1.0624000000000E-10
1.37660000000000	1.1957000000000E-11	1.9859000000000E-11
1.38200000000000	1.3677000000000E-12	2.4608000000000E-12
1.38650000000000	1.9462000000000E-13	3.7559000000000E-13
1.39040000000000	3.5945000000000E-14	8.6488000000000E-14
1.39360000000000	9.1176000000000E-15	2.0868000000000E-14
1.39650000000000	3.2707000000000E-15	7.3546000000000E-15
1.39900000000000	5.6944000000000E-16	1.7355000000000E-15
1.34310000000000	1.7954000000000E-08	2.1271000000000E-08
1.35640000000000	1.5937000000000E-09	1.9381000000000E-09
1.36670000000000	1.6558000000000E-10	2.1661000000000E-10
1.37480000000000	2.1771000000000E-11	3.5292000000000E-11
1.38120000000000	1.9134000000000E-12	3.4220000000000E-12
1.38650000000000	1.9462000000000E-13	3.7559000000000E-13
1.39090000000000	2.8104000000000E-14	6.9025000000000E-14
1.39460000000000	6.7571000000000E-15	1.4517000000000E-14
1.39770000000000	1.5751000000000E-15	4.2342000000000E-15
1.40030000000000	1.5289000000000E-16	5.6185000000000E-16

area-x pol	area-y pol	power-x pol
3.6841000000000E-08	3.9788000000000E-08	8.8306000000000E-01
3.4737000000000E-08	3.8713000000000E-08	8.9844000000000E-01
3.3018000000000E-08	3.7966000000000E-08	9.0951000000000E-01
3.1559000000000E-08	3.7409000000000E-08	9.1756000000000E-01
3.0278000000000E-08	3.6935000000000E-08	9.2341000000000E-01
2.9122000000000E-08	3.6461000000000E-08	9.2759000000000E-01
2.8046000000000E-08	3.5911000000000E-08	9.3046000000000E-01
2.7018000000000E-08	3.5221000000000E-08	9.3223000000000E-01
2.6010000000000E-08	3.4337000000000E-08	9.3303000000000E-01
2.4994000000000E-08	3.3215000000000E-08	9.3295000000000E-01
1.4713000000000E-08	1.5513000000000E-08	8.6705000000000E-01
1.7243000000000E-08	1.8848000000000E-08	8.8960000000000E-01
1.9984000000000E-08	2.2651000000000E-08	9.0484000000000E-01
2.2901000000000E-08	2.6897000000000E-08	9.1535000000000E-01
2.5959000000000E-08	3.1532000000000E-08	9.2263000000000E-01
2.9122000000000E-08	3.6461000000000E-08	9.2759000000000E-01
3.2341000000000E-08	4.1537000000000E-08	9.3080000000000E-01
3.5557000000000E-08	4.6560000000000E-08	9.3257000000000E-01
3.8692000000000E-08	5.1277000000000E-08	9.3310000000000E-01
4.1647000000000E-08	5.5396000000000E-08	9.3248000000000E-01

power-y pol	sens-x pol	sens y-pol	EML-x pol
8.7675000000000E-01	0.9329255263745	0.9271999408459	0.0084470000000
8.9027000000000E-01	0.9411912947252	0.9365620320070	0.0080585000000
9.0039000000000E-01	0.9464342664517	0.9426349901467	0.0078398000000
9.0802000000000E-01	0.9496612244898	0.9464878686619	0.0077449000000
9.1375000000000E-01	0.9514855235469	0.9488004341534	0.0077463000000
9.1797000000000E-01	0.9522685078080	0.9500020194735	0.0078305000000
9.2096000000000E-01	0.9522545585586	0.9502684119678	0.0079895000000
9.2289000000000E-01	0.9515286083956	0.9498899253731	0.0082231000000
9.2387000000000E-01	0.9502277845514	0.9487308270677	0.0085331000000
9.2395000000000E-01	0.9482420728652	0.9469542530379	0.0089292000000
8.6318000000000E-01	0.9233620818959	0.9166934703298	0.0089078000000
8.8243000000000E-01	0.9365599051008	0.9313122972574	0.0082735000000
8.9607000000000E-01	0.9443427899456	0.9401278993195	0.0079231000000
9.0590000000000E-01	0.9488261916928	0.9454444282805	0.0077629000000
9.1297000000000E-01	0.9512340085675	0.9485480741384	0.0077404000000
9.1797000000000E-01	0.9522685078080	0.9500020194735	0.0078305000000
9.2132000000000E-01	0.9521907643542	0.9502739233590	0.0080205000000
9.2328000000000E-01	0.9511919264473	0.9495549978488	0.0083101000000
9.2401000000000E-01	0.9494138721697	0.9479874078844	0.0087046000000
9.2357000000000E-01	0.9467478907479	0.9455985146040	0.0092198000000

EML-y pol	confinement -x pol	confinement-y pol
0.0124920000000	1.269091276970E-03	1.536000299299E-03
0.0119810000000	1.822752857721E-04	2.280282074949E-04
0.0115990000000	3.257570770011E-05	4.638453849326E-05
0.0113280000000	5.655480553410E-06	9.393007302013E-06
0.0111600000000	6.966631103801E-07	1.253453668219E-06
0.0110860000000	1.062142187683E-07	2.049789252246E-07
0.0111040000000	2.092485236120E-08	5.034771542678E-08
0.0112150000000	5.639406343205E-09	1.290724879025E-08
0.0114200000000	2.141988564334E-09	4.816543582491E-09
0.0117270000000	3.936456924514E-10	1.199726220935E-09
0.0129870000000	9.798428135684E-03	1.160868691512E-02
0.0122790000000	8.697646719305E-04	1.057721597960E-03
0.0117600000000	9.036558598121E-05	1.182153012404E-04
0.0114000000000	1.188156282399E-05	1.926067315164E-05
0.0111800000000	1.044241528062E-06	1.867562720303E-06
0.0110860000000	1.062142187683E-07	2.049789252246E-07
0.0111150000000	1.533780908573E-08	3.767051921943E-08
0.0112670000000	3.687699607644E-09	7.922679138116E-09
0.0115480000000	8.596136881205E-10	2.310822346670E-09
0.0119710000000	8.343999541410E-11	3.066306587966E-10

Total loss-x pol	Total loss-y pol	v para-x pol
9.716091276970E-03	1.402800029930E-02	2.49419693152896E+00
8.240775285772E-03	1.220902820749E-02	2.53440569315868E+00
7.872375707700E-03	1.164538453849E-02	2.56655464292903E+00
7.750555480553E-03	1.133739300730E-02	2.59152053884293E+00
7.746996663110E-03	1.116125345367E-02	2.61035539701804E+00
7.830606214219E-03	1.108620497893E-02	2.62346447579643E+00
7.989520924852E-03	1.110405034772E-02	2.63180993955040E+00
8.223105639406E-03	1.121501290725E-02	2.63404999971379E+00
8.533102141989E-03	1.142000481654E-02	2.63607182494368E+00
8.929200393646E-03	1.172700119973E-02	2.62787952486318E+00
1.870622813568E-02	2.459568691512E-02	2.45450810754084E+00
9.143264671930E-03	1.333672159796E-02	2.51078302230383E+00
8.013465585981E-03	1.187821530124E-02	2.55354411798181E+00
7.774781562824E-03	1.141926067315E-02	2.58396573568544E+00
7.741444241528E-03	1.118186756272E-02	2.60582706292983E+00
7.830606214219E-03	1.108620497893E-02	2.62346447579643E+00
8.020515337809E-03	1.111503767052E-02	2.63275560413321E+00
8.310103687700E-03	1.126700792268E-02	2.63522464174668E+00
8.704600859614E-03	1.154800231082E-02	2.63500387379131E+00
9.219800083440E-03	1.197100030663E-02	2.62333295964752E+00

v para-y pol	NA- x pol	NA- y pol
2.35740537948648E+00	4.03436223998823E-01	3.90569406299910E-01
2.40205946589047E+00	3.81558345859167E-01	3.64166584989110E-01
2.43822837222105E+00	3.61818600285830E-01	3.40334260922009E-01
2.46624586949875E+00	3.44072604933766E-01	3.18993193642361E-01
2.48763624933271E+00	3.28157755313603E-01	3.00042853884529E-01
2.50454686744271E+00	3.13898375904781E-01	2.83357897488349E-01
2.51294442321687E+00	3.01170914006819E-01	2.68838758849251E-01
2.52298715614126E+00	2.89857115909454E-01	2.56389898671562E-01
2.52170506763323E+00	2.79873069202713E-01	2.45931744459089E-01
2.51735093618773E+00	2.71180197513859E-01	2.37409195930695E-01
2.31703365642395E+00	4.21740648079128E-01	4.12618926075902E-01
2.37625459160117E+00	3.94758890989248E-01	3.80107596893318E-01
2.42152856930404E+00	3.70671162148655E-01	3.51016846167762E-01
2.45650783574507E+00	3.49330655696161E-01	3.25300686622165E-01
2.48570766550421E+00	3.30494470534832E-01	3.02806643497468E-01
2.50454686744271E+00	3.13898375904781E-01	2.83357897488349E-01
2.51581158030267E+00	2.99338836565820E-01	2.66792252975454E-01
2.51998144464752E+00	2.86644910602908E-01	2.52963679551883E-01
2.52000077356471E+00	2.75706288423231E-01	2.41760515402649E-01
2.51773821663984E+00	2.66464736664882E-01	2.33105886247125E-01

spot-x pol	spot-y pol	nonlinear- x pol
1.562239080249E-04	1.622086529764E-04	1.510624071193E-12
1.546461481784E-04	1.601410177107E-04	1.484764360789E-12
1.534366276398E-04	1.585501742233E-04	1.466692716196E-12
1.525274215803E-04	1.573659234442E-04	1.453825027582E-12
1.518581402690E-04	1.564885336399E-04	1.444614734850E-12
1.514004975978E-04	1.558106184247E-04	1.438104865628E-12
1.511125720664E-04	1.554789888528E-04	1.433669037151E-12
1.510357347189E-04	1.550866640422E-04	1.430946968353E-12
1.509665443034E-04	1.551364929439E-04	1.429720043630E-12
1.512478467134E-04	1.553062771835E-04	1.429842641415E-12
9.714284378277E-05	1.010368529248E-04	2.500091113546E-12
1.076981758134E-04	1.116839974431E-04	2.165971223022E-12
1.184005739337E-04	1.225198093037E-04	1.916541266964E-12
1.292921870536E-04	1.335001799382E-04	1.722305129186E-12
1.403240982141E-04	1.445231246689E-04	1.566322361076E-12
1.514004975978E-04	1.558106184247E-04	1.438104865628E-12
1.627016584842E-04	1.673177801588E-04	1.330777825526E-12
1.742255974923E-04	1.790810770512E-04	1.239701899053E-12
1.858499353260E-04	1.910188883846E-04	1.161560390098E-12
1.979912272607E-04	2.030730299173E-04	1.093960192176E-12

	birefringence
1.431115041109E+01	-8.29999999999997E-03
1.448819634833E+01	-6.70000000000015E-03
1.462736070719E+01	-5.50000000000006E-03
1.473266665647E+01	-4.59999999999994E-03
1.481164698502E+01	-3.89999999999979E-03
1.487324032259E+01	-3.30000000000008E-03
1.490355392470E+01	-2.90000000000012E-03
1.493957186162E+01	-2.39999999999996E-03
1.493498781948E+01	-2.19999999999998E-03
1.491938902990E+01	-1.90000000000001E-03
1.414584701309E+01	-9.70000000000004E-03
1.438660791062E+01	-7.60000000000005E-03
1.456356533735E+01	-6.09999999999999E-03
1.469630710778E+01	-4.90000000000013E-03
1.480457541526E+01	-3.90000000000001E-03
1.487324032259E+01	-3.30000000000008E-03
1.491386282487E+01	-2.80000000000014E-03
1.492881859469E+01	-2.39999999999996E-03
1.492888781849E+01	-2.09999999999999E-03
1.492077840509E+01	-1.70000000000003E-03
